# Intraoperative Tension Pneumothorax in a Patient With Remote Trauma and Previous Tracheostomy

**DOI:** 10.1177/2324709616636397

**Published:** 2016-02-29

**Authors:** Ana Mavarez-Martinez, Suren Soghomonyan, Gurneet Sandhu, Demicha Rankin

**Affiliations:** 1 The Ohio State University Wexner Medical Center, Columbus, OH, USA

**Keywords:** intraoperative pneumothorax, thoracic trauma, occult pneumothorax, previous tracheostomy

## Abstract

Many trauma patients present with a combination of cranial and thoracic injury. Anesthesia for these patients carries the risk of intraoperative hemodynamic instability and respiratory complications during mechanical ventilation. Massive air leakage through a lacerated lung will result in inadequate ventilation and hypoxemia and, if left undiagnosed, may significantly compromise the hemodynamic function and create a life-threatening situation. Even though these complications are more characteristic for the early phase of trauma management, in some cases, such a scenario may develop even months after the initial trauma. We report a case of a 25-year-old patient with remote thoracic trauma, who developed an intraoperative tension pneumothorax and hemodynamic instability while undergoing an elective cranioplasty. The intraoperative patient assessment was made even more challenging by unexpected massive blood loss from the surgical site. Timely recognition and management of intraoperative pneumothorax along with adequate blood replacement stabilized the patient and helped avoid an unfavorable outcome. This case highlights the risks of intraoperative pneumothorax in trauma patients, which may develop even months after injury. A high index of suspicion and timely decompression can be life saving in this type of situation.

## Introduction

Intraoperative tension pneumothorax is a dangerous complication of mechanical ventilation, which, if undiagnosed, is associated with a high mortality rate.^1^ In adult surgical patients, the most common etiologies of pneumothorax include thoracic trauma or iatrogenic injuries, such as complications of central line placement, regional blocks, or mechanical ventilation.^2^ The tension pneumothorax during general anesthesia and mechanical ventilation manifests as changes in pulmonary compliance, increases in airway pressures, arterial hypotension, arrhythmias, and hypoxia.^3^

Patients with chest trauma may initially present with adequate ventilation and oxygenation without any radiological evidence of lung trauma. However, these patients are at risk of developing tension pneumothorax under positive-pressure ventilation.^4-6^

Some of the trauma patients remain intubated for prolonged periods of time and may eventually need tracheostomy to facilitate their respiratory care.^4^ In some cases, the patients are discharged from the hospital with the tracheotomy tube in place, with an increased risk of late complications (tracheal stenosis, pneumonia, etc) requiring chronic treatment, including bronchoscopies, tracheal dilations, and other measures.^5^

Under such circumstances, application of positive pressure in a patient with a history of repetitive tracheobronchial manipulation poses a risk of tissue rupture and dissection along tissue planes. Positive-pressure ventilation in such patients should be performed with caution, and the personnel should be prepared to treat the possible complications, including tension pneumothorax. When pneumothorax is diagnosed, needle decompression can be used as an initial treatment, followed by more definitive treatment with a tube thoracostomy.^6^

We present a patient with remote trauma and tracheal stenosis who developed tension pneumothorax during elective cranioplasty 9 months after the initial trauma. An informed consent was obtained from the patient to present this case.

## Case Presentation

A 25-year-old man with a medical history of motor vehicle accident 9 months earlier was scheduled for an elective cranioplasty. At the time of trauma, his injuries included acute traumatic subdural hematoma with severe cerebral contusion, multiple rib fractures, and traumatic pneumothorax, which were emergently managed with decompressive craniotomy with hematoma evacuation and tube thoracostomy. Subsequently, the patient developed respiratory failure requiring prolonged mechanical ventilation and tracheotomy. The postoperative course was complicated by development of a midtracheal stricture, necessitating staged dilations and bronchoscopies. The patient was discharged to a chronic care facility for continuous treatment and rehabilitation.

The patient underwent an uneventful flexible bronchoscopy, with replacement of the Shiley extended-length cuffless tracheostomy tube, 15 days prior to the elective cranioplasty. A follow-up chest X-ray did not demonstrate any complications, and the patient was discharged to the chronic care facility.

On the day of surgery, the patient was admitted to hospital and, following the standard preparations, transferred to the operating room. General anesthesia was induced with propofol and rocuronium. The cuffless tracheostomy tube was atraumatically exchanged for a 6.0-wire-reinforced cuffed endotracheal tube, under bronchoscopic guidance. Bilateral breath sounds and end tidal CO_2_ (PETCO_2_) were verified, and the endotracheal tube was sutured to the anterior chest wall skin.

Mechanical ventilation was initiated using the pressure-controlled volume guaranteed mode, with a peak airway pressure of 22 cm H_2_O, tidal volume 550 mL (6 mL/kg), and positive end expiratory pressure 5 cm H_2_O. The hemoglobin saturation was stably maintained at 100%, with 0.6 FiO_2._ The PETCO_2_ was maintained at 34 mm Hg.

The radial artery was catheterized for invasive blood pressure (BP) monitoring, and two 18-gauge intravenous catheters were placed. The patient was positioned supine with shoulder elevation and significant rotation of the head to the left.

Approximately an hour after starting the surgery, the hemoglobin saturation gradually decreased to 92% to 95%. despite increasing the FiO_2_ to 1.0. Lung auscultation confirmed bilateral respiratory sounds. During the subsequent 2 to 3 hours, BP instability was noted. The changes were attributed to the hypovolemia caused by ongoing intraoperative blood loss. The arterial hypotension initially responded to crystalloid infusions and phenylephrine boluses; however, with time, the hemodynamic instability and hypoxemia progressed.

The surgical procedure was complicated by an estimated 1.5 L of blood loss, which obscured the clinical picture even more. In addition to crystalloids, a phenylephrine infusion was started to maintain adequate BP. Blood transfusion was initiated to replace the ongoing blood loss, and small boluses of epinephrine were administered to stabilize the BP. At the end of surgery, when the galea and skin were being closed, a sudden arterial hypotension to systolic BP 40 to 60 mm Hg took place, along with critical desaturation. The surgery was halted, and the surgical wound was closed with sterile drapes. The endotracheal tube security was assessed, and lungs were auscultated. Breath sounds were absent on the left side. Without hesitation, a 2-inch 14-gauge angiocatheter was inserted into the left anterior chest wall at the second intercostal space. There was an obvious and audible “whoosh” of air accompanied by a dramatic improvement of hemodynamics and blood oxygenation.

**Figure 1.**
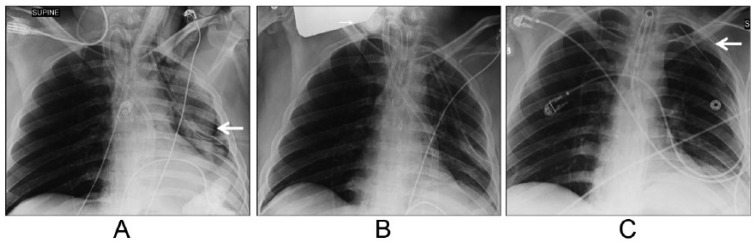
Results of patient’s X-ray examinations: (A) intraoperative chest X-ray demonstrating left pneumothorax; (B) intraoperative chest X-ray after left chest tube placement; (C) postoperative chest X-ray.

A STAT chest X-ray confirmed the presence of a left tension pneumothorax (Figure 1A), and a definitive treatment by thoracostomy was done. The correct positions of the endotracheal tube and thoracostomy catheter were verified (Figure 1B).

On completion of surgery, the patient was transferred to the critical care unit (CCU) under ventilatory assistance. A subsequent chest X-ray performed in the CCU revealed a 2-mm residual pneumothorax present along the lateral aspect of the left apical region and decreased lung volumes on the left side (Figure 1C). On postoperative day 4, the endotracheal tube was replaced with an XLT Shiley tracheostomy tube, and after 2 uneventful days, the patient was discharged to the chronic care facility.

## Discussion

Many patients who sustain severe traumatic brain injury (TBI), thoracic trauma, and posttracheostomy tracheal stenosis require chronic oxygen therapy,^7^ which may not be an indication of acute underlying pathology.

Our patient presented with oxygen therapy via a tracheal mask, and this was not an alarming preoperative finding. He had suffered a thoracic trauma 9 months earlier and had recovered from it. The patient’s main chronic problems were the sequelae of severe TBI and posttracheostomy stenosis. Interestingly, during a post hoc review of his medical record, it was noted that he was placed on 5 L/min tracheal mask oxygen during admission for his cranioplasty. He did not have oxygen requirements before the bronchoscopy. His preoperative blood oxygenation at room air was 100%. No objective data existed to suspect the presence of a pneumothorax in the patient before surgery. Several tentative mechanisms may explain the development of intraoperative tension pneumothorax in our patient.

First, it is possible the thoracic trauma that the patient suffered 9 months earlier healed with “weak” zones or areas of chronic inflammation in the lungs, prone to disection once the patient was connected to a ventilator. At the time of trauma, the patient had suffered rib fractures and was diagnosed with pneumothorax. Presumably, a previously unrecognized visceral pleural laceration related to the original trauma caused an air leak, resulting in the tension pneumothorax.^8^

Plourde et al^9^ studied a cohort of 450 patients with thoracic trauma. They reported a delayed pneumothorax in 0.9% of the patients during the first 14 days. The presence of at least 1 rib fracture on the X-ray represented a significant risk factor for this complication. According to Moore et al,^10^ patients receiving mechanical ventilation are at a higher risk of developing delayed pneumothorax resulting from previous chest trauma. They observed 448 blunt trauma patients identified with thoracic lesions. In the group of 73 patients who were under mechanical ventilation, 14% developed pneumothorax while on positive-pressure ventilation. Both these studies described patients who developed pneumothorax in the early stage of thoracic trauma. In our patient, the tension pneumothorax developed 9 months after the initial trauma, when all lesions had presumably healed.

Second, the bronchoscopy performed 15 days prior to elective surgery could have created a subclinical pneumothorax. Under this assumption, the patient would have remained asymptomatic, given that he was breathing spontaneously. If so, this subclinical pneumothorax could have transitioned to tension pneumothorax with positive-pressure ventilation during the cranioplasty.^11^ Even though flexible bronchoscopy is considered a safe procedure, there are reports indicating complication rates ranging from <0.1% to 11%.^12^ The most common complication of the procedure is bleeding, whereas pneumothorax constitutes 0.07% to 0.16% of complications.

Third, replacement of the cuffless tracheostomy tube with an armored cuffed endotracheal tube, performed under bronchoscopic guidance could have created a small tear in the mucosa, serving as an entrance for air under positive-pressure ventilation. Tracheal rupture with tension pneumothorax has a low incidence of <1%, but it is known to be a potentially life-threatening complication.^13^

A typical mucosal laceration of the trachea will manifest as a progressive subcutaneous emphysema under positive-pressure ventilation, which was not the case with our patient. We consider this option less likely because the endotracheal tube was lubricated and passed easily. We cannot exclude the possibility of trauma to the preexisting, already weakened areas of mucosa during intubation, even though repetitive bronchoscopic evaluations, including intraoperative bronchoscopic examination, did not reveal any mucosal trauma in our patient.

The index of suspicion for a pneumothorax causing the clinical deterioration was relatively low in this patient, considering the following factors:

- the 9-month period between initial thoracic trauma and elective surgery;
- no attempts at central venous access;
- the surgical procedure did not involve the chest; and
- no pneumothorax was revealed on postprocedural chest X-ray films taken after bronchoscopic replacement of the tracheostomy tube 15 days prior to surgery.

In addition to the low index of suspicion, there were additional factors obscuring the clinical picture. The gradual development (over several hours) of clinical symptoms masked the initial presentation. More important, the unexpected high-volume blood loss with tachycardia during surgery and hemodynamic instability initially suggested anemia and hypovolemia rather than pneumothorax as the cause of deterioration.^14^ It must also be taken into consideration that auscultative assessment of the lungs, particularly the left lung, was challenging because of the limited access to the patient’s chest and his position on the operating table with a slight tilt toward the left. Another important consideration was reluctance to interrupt the surgery in an actively bleeding patient.

Thus, despite the complexity and presence of multiple factors obscuring the clinical picture in our patient, the correct diagnosis of tension pneumothorax was made and definitive life-saving measures were taken. Progressive, otherwise unexplained, hypoxemia and hemodynamic instability unresponsive to therapy and disproportionate to the intraoperative blood loss were the key factors indicating the possibility of a tension pneumothorax in our patient.

## Conclusion

The intraoperative progression of a simple or occult pneumothorax into a tension pneumothorax can be a devastating clinical scenario. Even patients with remote chest trauma remain at a higher risk of developing such a complication under mechanical ventilation. The effective management of an intraoperative tension pneumothorax requires prompt recognition of the problem and immediate drainage of the intrapleural air by needle decompression and thoracostomy. The intraoperative presentation of pneumothorax may be masked by many factors, as in our patient; nevertheless, progressive hypoxia and hemodynamic instability should put tension pneumothorax on the top of the list of differential diagnoses.
